# Nanofluids of Amphiphilic Kaolinite-Based Janus Nanosheets for Enhanced Oil Recovery: The Importance of Stable Emulsion

**DOI:** 10.3390/polym15112515

**Published:** 2023-05-30

**Authors:** Yixuan Mao, Alain Luigi Lanzon, Botuo Zheng, Zhengxiao Xu, Jiatong Jiang, David Harbottle, Kai Yu, Mingfeng Chen, Yu Sheng, Huagui Zhang

**Affiliations:** 1 Fujian Key Laboratory of Polymer Materials, College of Chemistry and Materials Science, Fujian Normal University, Fuzhou 350007, Chinazhengbotuo@fjnu.edu.cn (B.Z.); cmfjnu@fjnu.edu.cn (M.C.); 2 School of Petroleum Engineering, Changzhou University, Changzhou 213164, China; 3 School of Chemical and Process Engineering, University of Leeds, Leeds LS2 9JT, UK; 4 School of Energy and Power Engineering, Jiangsu University, Zhenjiang 212013, China; victoryu66@ujs.edu.cn

**Keywords:** nanofluid, Janus nanosheets, clay, enhanced oil recovery, rheology, poly(N-Isopropylacrylamide)

## Abstract

To meet the increasing global demand for energy, better recovery of crude oil from reservoirs must be achieved using methods that are economical and environmentally benign. Here, we have developed a nanofluid of amphiphilic clay-based Janus nanosheets via a facile and scalable method that provides potential to enhance oil recovery. With the aid of dimethyl sulfoxide (DMSO) intercalation and ultrasonication, kaolinite was exfoliated into nanosheets (KaolNS) before being grafted with 3-methacryloxypropyl-triemethoxysilane (KH570) on the Alumina Octahedral Sheet at 40 and 70 °C to form amphiphilic Janus nanosheets (i.e., KaolKH@40 and KaolKH@70). The amphiphilicity and Janus nature of the KaolKH nanosheets have been well demonstrated, with distinct wettability obtained on two sides of the nanosheets, and the KaolKH@70 was more amphiphilic than the KaolKH@40. Upon preparing Pickering emulsion in a hydrophilic glass tube, the KaolKH@40 preferentially stabilized emulsions, while the KaolNS and KaolKH@70 tended to form an observable and high-strength elastic planar interfacial film at the oil–water interface as well as films climbing along the tube’s surface, which were supposed to be the result of emulsion instability and the strong adherence of Janus nanosheets towards tube’s surface. Subsequently, the KaolKH was grafted with poly(N-Isopropylacrylamide) (PNIPAAm), and the prepared thermo-responsive Janus nanosheets demonstrated a reversible transformation between stable emulsion and the observable interfacial films. Finally, when the samples were subjected to core flooding tests, the nanofluid containing 0.01 wt% KaolKH@40 that formed stable emulsions showed an enhanced oil recovery (EOR) rate of 22.37%, outperforming the other nanofluids that formed observable films (an EOR rate ~13%), showcasing the superiority of Pickering emulsions from interfacial films. This work demonstrates that KH-570-modified amphiphilic clay-based Janus nanosheets have the potential to be used to improve oil recovery, especially when it is able to form stable Pickering emulsions.

## 1. Introduction

Despite the growing transition to renewable energy sources, for the foreseeable future, crude oil will remain a significant contributor to the energy matrix. Furthermore, beyond fuel, crude oil is also a valuable chemical feedstock for a range of materials [1], with new materials from crude oil being explored. In crude oil recovery, it is said that after the primary (i.e., driven by pressure difference between wells and reservoirs) and secondary (i.e., water flooding) stages of oil extraction, roughly 70% of the oil initially in place (OIIP) is left behind [2], making the tertiary recovery stage, widely known as enhanced oil recovery (EOR), extremely important to maximize the amount of recoverable oil from the reservoir [3,4].

Nanofluids, namely, fluids containing nanoparticles, have attracted much research interest in the past decades as oil displacement agents for enhanced oil recovery applications and have exhibited advantages in terms of high oil recovery, and stable fluctuation of differential pressure, etc., as compared to conventional secondary or tertiary recovery techniques such as water flooding or surfactant flooding [4,5,6,7,8,9,10,11]. The main mechanisms of oil displacement by nanofluid flooding are argued to include the reduction in oil–water interfacial tension, alteration of rock surface wettability, generation of structural disjoining pressure, as well as plugging and profile control, etc. [4]. However, the efficiency of nanofluid flooding at low concentration (0.01 wt% or less) of nanoparticles is reported to be low, below 5% in a saline environment (2 wt% or higher NaCl content) [12], with the optimal mass concentration of Al_2_O_3_ and SiO_2_ nanofluids needing to be as high as 1.0% and 0.5%, respectively [13]. The use of high concentration nanofluids is neither economical nor environmentally friendly. In addition, it is worth noting that, to date, the most commonly concerned nanoparticles in nanofluid flooding are spherical particles, particularly the silica nanoparticles thanks to their stable properties and low price [4], with non-isotropic particles rarely focused.

Recently, Luo et al. [12] reported a simple nanofluid of graphene-based Janus amphiphilic nanosheets for EOR with efficiency of about 15.2% at a nanofluid concentration of 0.005 wt%, which was comparable to chemical methods whilst being more economically and environmentally beneficial to the petroleum industry. Likewise, based on a nanofluid of carboxyl/alkyl composite silica-based amphiphilic Janus nanosheets, Yin et al. [14] showed an enhanced oil recovery factor by as much as 18.31% at a nanosheet concentration of 0.005 wt%. They attributed the high EOR efficiency of Janus nanosheets at low concentrations to their outstanding interfacial activities that enable the formation of a macroscopically observable and high-strength elastic interfacial film at the oil–water interface and a climbing film along the solid walls that promotes the detachment of crude oil from the rock surface.

In fact, upon nanofluid flooding, not matter containing homogenous or heterogeneous nanoparticles, Pickering emulsion can be produced when nanoparticles are effectively anchored at the oil–water interface, and this kind of emulsion is far more stable due to the almost irreversible adsorption of the particles at the interface with extremely high detachment energies of more than 10,000 kBT [15,16,17]. It is believed that the oil–water interfaces in Pickering emulsions have better viscoelastic properties than those of traditional surfactant-stabilized emulsions and act a key role in minimizing the coalescence rate and/or the Ostwald ripening of the emulsions [18]. This stabilizing mechanism is essential to maintain emulsion stability under extreme reservoir conditions (high temperature, pressure, and salinity), as the particle-laden film strongly resists the collapse of the thin-liquid film and droplet coalescence. Emulsification of oil–water has been found to have a higher increase on oil recovery factor than those with no emulsions present [19]. The emulsion drops are reported to lead to more favorable fluidity of the oil in porous media [20], while they supply additional pressure to the porous medium, which leads to the recovery of the residual oil in the pores via a piston effect when the pressure was increased [21]. That is to say, nanoparticle-stabilized emulsions can increase oil recovery, and, indeed, there have been already extensive studies using nanoparticle-stabilized emulsions as EOR agents [22].

Back to the nanofluid of Janus nanosheets, aside from forming a macroscopically observable interfacial film and climbing films that are argued to be vital for EOR, it is also possible that Janus-nanosheets-stabilized emulsions are being produced during nanofluid flooding, but its effect has yet been focused on to date. The specific EOR mechanism of Janus nanosheets, in fact, still remains elusive. Moreover, the Janus nanoparticles used for EOR application thus far involved complex synthesis methods, and the feedstocks were not inexpensive [12].

**Figure 1:**
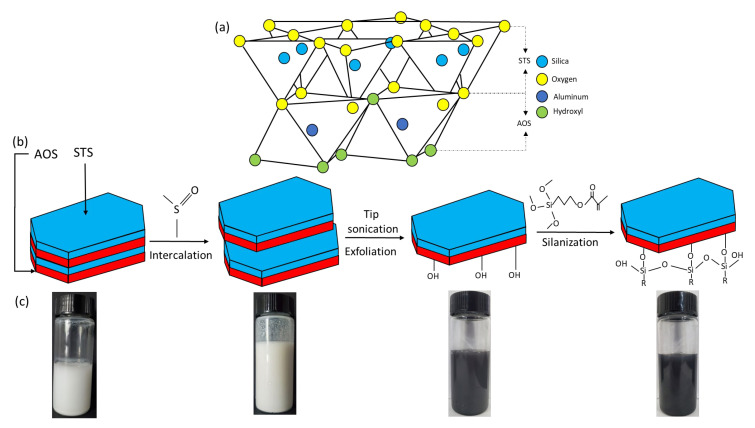
(**a**) Schematic of crystal structure of a single layer of kaolinite; (**b**) schematic of the multi-staged process of chemical modification from pristine kaolinite to Janus nanosheets with KH 570 molecules on AOS side; (**c**) the corresponding photos of samples in each stage.

Kaolinite is abundantly found in soils, sediments, and sedimentary rocks and consists of a stacked layer of nanosheets, providing the clay with a Janus characteristic. One side consists of an Alumina Octahedral Sheet (AOS), and the other is occupied by a Silica Tetrahedral Sheet (STS), linked by sharing a plane of oxygen atoms (Figure 1a) [23]. Despite its relative abundance and low cost, to our knowledge, the exploitation of the Janus characteristic of kaolinite has not yet been considered for enhanced oil recovery.

In the present study, we have prepared an amphiphilic Janus nanosheet based on exfoliated kaolinite by grafting a silane coupling agent and investigated its interfacial behavior at oil–water interface and its potential to be used for enhanced oil recovery, whether to form a stable interfacial film or to produce a stable Pickering emulsion to enhance oil recovery. The hidden Janus characteristic of kaolinite can greatly simplify the whole synthesis process to produce amphiphilic Janus nanoparticles (AJNPs) in a scalable way. The size, surface properties, and morphologies, etc., of the Janus nanosheets have been characterized by a series of techniques, including using a scanning electron microscope (SEM), light scattering, zeta potential, and contact angle measurements, etc. Furthermore, by simply tweaking some of the processes, the amphiphilicities of the Janus nanosheets can be varied, and their effects on EOR, especially the importance of Janus-nanosheets-stabilized emulsions, were evaluated based on core flooding experiments based on hydrophilic sandstones. It was demonstrated to achieve an enhanced EOR rate once the interfacial behavior of the Janus nanosheets was optimized to form stable emulsions, giving a promising application potential on crude oil recovery with an economical and green route.

## 2. Experimental Section

### 2.1. Materials

Kaolinite was purchased from Aladdin (China). Ethanol (≥99.7%), dimethyl sulfoxide (DMSO, analytical grade, ≥99.5%), acetic acid (≥99.5%), anhydrous sodium acetate (≥99%), sodium chloride (NaCl, ≥99.5%), 3-methacryloxypropyltriemethoxysilane (KH-570, ≥98%), N-isopropylacrylamide (NIPAAm, 98%), potassium persulfate (KPS, ≥99.5%), and toluene (≥99.5%) were all purchased from Sinopharm Chemical Reagent Co., Ltd. (Shanghai, China). The chemicals were used as received.

### 2.2. Methods

#### 2.2.1. Intercalation and Exfoliation of Kaolinite

Pristine kaolinite (Kaol) was dispersed in DMSO/water (10/1 *v*/*v*, 1 g Kaol/1 g water) mixture before being subjected to heating at 80 °C for 24 h with gentle magnetic stirring. The intercalated kaolinite was then collected by centrifuging at 3400 rpm for 5 min and washed with water and ethanol three times. The intercalated kaolinite was then dispersed in water and subjected to tip sonication (ultrasonic horn 6 mm, power 650 W, ratio 70%, BZLON-650Y) for 2.5 h at a 2 s-by-3 s on–off pulse under an ice bath that prevented heating of the mixture. Afterwards, the suspension was centrifuged at the same conditions to settle the unexfoliated particles, and the supernatant with the exfoliated kaolinite nanosheets (KaolNS) present was thus collected. The exfoliation of Kaol into nanosheets was accompanied with a clear change in suspension color from white to dark blue (see Figure 1c) due to the surface plasmon resonance as a consequence of the reduction in particle size [24] as well as the presence of various hydrolysable metal ions released from the interlayer of Kaol (verified by the zeta potential measurements shown later).

#### 2.2.2. Silanization of Kaolinite Nanosheets

The kaolinite nanosheets (KaolNS) in water (1.5 g/L) were ultrasonicated for 30 min, and the pH value was adjusted to ~3 with the addition of ~1 mL acetic acid buffer. The KH 570 silane coupling agent (SCA) was then added dropwise by mechanical pipette (Pipet-LiteTM PL+, Mettler Toledo) in the KaolNS suspension at a ratio of 1 g KaolNS/2 mL SCA under stirring. After 5 min of hydrolyzation at room temperature, with stirring, the system was allowed to react at 70 °C (or 40 °C) for 3 h, with stirring and reflux. The modified KaolNS-KH 570 nanosheets (KaolKH) were collected by centrifuging at 9000× *g* rpm for 5 min and washed with water three times until the solution pH was neutral. The as-obtained KaolKH was placed in 20 mL distilled water and stored as stock suspensions for further use. The KaolKH concentrations were measured by dry mass measurement (i.e., by drying in an oven 1 mL of the prepared samples) to be around 1.5 g/L. Note that such KaolKH Janus nanosheets could be produced in gram scale, showing a feasibility of scale up for practical applications.

#### 2.2.3. Grafting Polymerization of NIPAAm onto KaolKH Janus Nanosheets

In total, 1 g of KaolKH was dispersed in 85 mL ultrapure water and ultrasonicated for 30 min. A total of 2 g NIPAAm was dissolved in 20 mL ultrapure water. Then, both the KaolKH dispersion and the NIPAAm solution were added to a three-neck round-bottom flask, and the mixture was bubbled with nitrogen for 30 min under stirring. Subsequently, the mixture was heated to 70 °C and purged with nitrogen. Polymerization was initiated by injecting a KPS aqueous solution (0.11 g dissolved beforehand in 5 mL ultrapure water) to the system via a long-needle syringe. The reaction was carried out for 4 h at 70 °C under continuous nitrogen flow, reflux, and stirring. The obtained product was centrifuged at 9000× *g* rpm for 5 min and was cleaned with ultrapure water three times to remove the free PNIPAAm chains. The obtained particles of PNIPAAm-grafted Kaol amphiphilic Janus nanosheets were named KaolNP. For comparison, pure PNIPAAm was also synthesized under identical conditions but in the absence of KaolKH particles.

### 2.3. Characterizations

#### 2.3.1. X-ray Diffraction (XRD)

The powder XRD patterns were collected using a X’Pert PXRD X-ray diffractometer (Panaco X-ray Analysis Instruments, Netherlands) with CuKα (λ = 0.154 nm) radiation at a scanning rate of 0.40°/min in the 2θ range of 5 to 80°, operating at 40 kV and 40 mA.

#### 2.3.2. Fourier Transform Infrared (FT-IR)

The infrared spectra were collected using Nicolet 5700 FTIR Spectrometer (Thermo Nicolet Co., Waltham, MA, USA). The samples were ground with KBr at a ratio of 100:1 (KBr: Sample). The data between 400 to 4000 cm^−1^ were collected with 4 cm^−1^ resolution and 16 scans.

Thermogravimetric Analysis (TGA). The TGA curve were collected using a TGA/SDTA851e (Mettler-Toledo, Zurich, Switzerland). The samples were heated from 30 °C to 800 °C at a heating rate of 10 °C/min under a flowing nitrogen atmosphere of 50 mL/min.

#### 2.3.3. Zeta Potential and Dynamic Light Scattering (DLS)

To measure the zeta potential and particle size, all samples were diluted to a concentration around 0.1 wt% and measured using a Zetasizer Nano ZS (Malvern Panalytical, Westborough, MA, USA). The zeta potential was measured from pH 1 to pH 13 in the presence of background electrolyte (0.01 M NaCl), and the size was measured at pH 7 with a dynamic light scattering angle of 173°.

#### 2.3.4. Atomic Force Microscopy (AFM)

Samples for AFM measurement were prepared by depositing one drop of ~10 ppm exfoliated or pristine kaolinite suspension on a clean silicon wafer and dried at room temperature. The imaging was acquired in tapping mode using a Bruker MultiMode 8 AFM microscope equipped with a NCHV silicon cantilever (Bruker, Billerica, MA, USA) with a force constant of ~42 N m^−1^ and a resonance frequency of ~320 kHz. Height profiles were collected by scanning at 1 Hz over the samples.

#### 2.3.5. Scanning Electron Microscopy (SEM)

SEM characterizations were performed in a Phenomenon LE microscope (Phenomenon, LE, Netherlands) at an acceleration voltage of 5 kV. Before measurement, monocrystalline silicon of high purity was first cleaned with distilled water before a droplet of ~10 ppm sample was deposited and allowed to dry at room temperature.

#### 2.3.6. Water Contact Angle

The water contact angle (WCA) measurement was performed using a surface tensiometer (DSA25, KRüSS, Germany). Smooth glass slides were used as the substrate and the glass surfaces were cleaned with acetone, ethanol, and distilled water. The unmodified samples (c.a. 1.5 g/L) were deposited by pipette (1 mL) onto the clean slide and allowed to dry at room temperature before 1 µL of water was added as a single droplet to measure the contact angle. At least five sessile drop measurements were conducted at different locations to achieve statistical confidence. For the Janus nanosheets, the WCA measurement was conducted on either side of the nanosheets based on interfacial films covering glass slides from different faces of the nanosheets (see details in Section 2.4.3).

#### 2.3.7. Optical Microcopy

All images were collected using an optical microscope (Shenzhen Ai Ke Xue, co. Ltd. Shenzhen, China). For samples of emulsion, to improve phase identification, a hydrophilic dye of color orange red (sugarman^®^, San Francisco, CA, USA) was used to stain the aqueous phase of the emulsion. The droplet size distribution was determined by image analysis using ImageJ software (Version 1.53c, USA).

#### 2.3.8. Rheology Measurements

Rheology analysis of Pickering emulsions prepared by Janus nanosheets was achieved using a Discovery Hybrid Rheometer HR 20 (TA instruments, New Castle, DE, USA) with a cone and plate geometry (i.e., 40 mm 1.00806° cone plate). Steady shear measurement was carried out in a shear rate range of 0.01–1000 s^−1^. Temperature ramp measurement was carried out in small amplitude oscillatory shear mode within the Linear Viscoelastic Region (LVR) determined beforehand, with a given angular frequency of 6.28319 rad/s and the temperature increasing from 25 °C to 45 °C at 5 °C/min.

### 2.4. Interfacial Study of KaolKH Janus Nanosheets at Oil–Water Interface

#### 2.4.1. Observation of Interfacial Phenomenon in Oil–Nanosheet–Water System

The water and oil phases were in brine, which was prepared at 0.1 wt% NaCl and toluene. This system was chosen as it approximately represented the interfacial tension of a crude oil–brine system [25], with interfacial tension values between 20–30 mN/m [26,27]. The toluene–brine volume ratio was fixed at 50/50 *v*/*v* (i.e., 3 mL for each fluid) and was added to a glass tube of radius 15 mm. Then, a given volume of the stock suspension of Janus nanosheets was injected into the brine solution at different concentrations from 0.005 wt% to 0.06 wt% relative to the aqueous phase. Afterwards, to simulate the hydrodynamic scenario in an oil reservoir, all the systems were subjected to a 30-s ultrasonication using a BioBlock Vibra-cell sonicator, equipped with an ultrasonic tip, and the phenomena (e.g., formation of interfacial films and/or emulsion, etc.) that happened at the oil–water interface were observed and recorded.

#### 2.4.2. Elasticity Testing of the Interfacial Film

1. Apparent testing and eye observation

For the samples where observable interfacial films formed at the oil–water interface, the elasticity of the interfacial film was apparently tested by poking with a plastic pipette. The interfacial film’s elasticity was also tested by releasing water and toluene droplets towards the interfacial film in a slow and aggressive manner.

2. Interfacial rheology measurements

To quantitatively assess the elasticity of the interfacial film, measurement of interfacial rheology at oil–water interface was performed using a Du Nouy ring (radius R = 19.8 mm) mounted in the HR-20 stress-control rheometer (TA Instruments, New Castle, DE, USA). A toluene/brine system containing a specific amount of KaolKH@70 was subjected to ultrasonication in a 27 mm glass tube and then transferred immediately to the PTFE trough mounted on Peltier heating system of the rheometer before the Du Noüy ring was lowered to pin the oil–water interface. Afterwards, a pre-shear (100 s^−1^ for 3 min) was given before strain amplitude sweep test was performed at 1 rad s^−1^. Dynamic frequency sweep from 0.1 to 100 rad s^−1^ was performed with a 0.1% shear strain. All measurements were performed at 25 °C.

#### 2.4.3. Contact Angle of the Interfacial Film

The Langmuir–Blodgett technique was used to obtain the interfacial film by vertically lifting a clean glass slide that was pre-positioned at the bottom of the vessel with its face parallel with the toluene/saline water interface whereby the intact interfacial film was transferred onto the glass slide with the hydrophobic side exposed to the air. Likewise, the interfacial film with its hydrophilic side exposed to air was obtained by vertically pressing a clean glass slide from the top onto the interfacial film formed by the Janus nanosheets at an air–water interface. The substrate was then observed under microscope for verification of the interfacial film, followed by drying under vacuum at 60 °C to remove any residual solvent before the WCA measurements, following the procedures of Section 2.3.6.

### 2.5. Core Flooding Test

The oil displacement experiments were conducted based on the hydrophilic core of sandstone that had a diameter of 2.54 cm, length of 4 cm, and a core volume of 21 cm^3^. The permeability and porosity (roughly 40%) of the core were first measured before saturating the core with 8.5 g of brine and then further saturated with 6.8, 6.8, 6.9, and 7.1 g of oil at an injection rate of 0.5 mL/min. After 8 pore volumes (PVs) of brine were injected to the core at a continuous flow rate of 0.5 mL/min, 6 PVs of nanofluid with a nanosheet concentration of 0.01 wt% were injected at the same flow rate. The volumes of produced oil were recorded, and the pressure difference was measured during flooding.

## 3. Results and Discussion

### 3.1. Preparation and Characterizations of Kaolinite-Based Amphiphilic Janus Nanosheets

**Figure 2:**
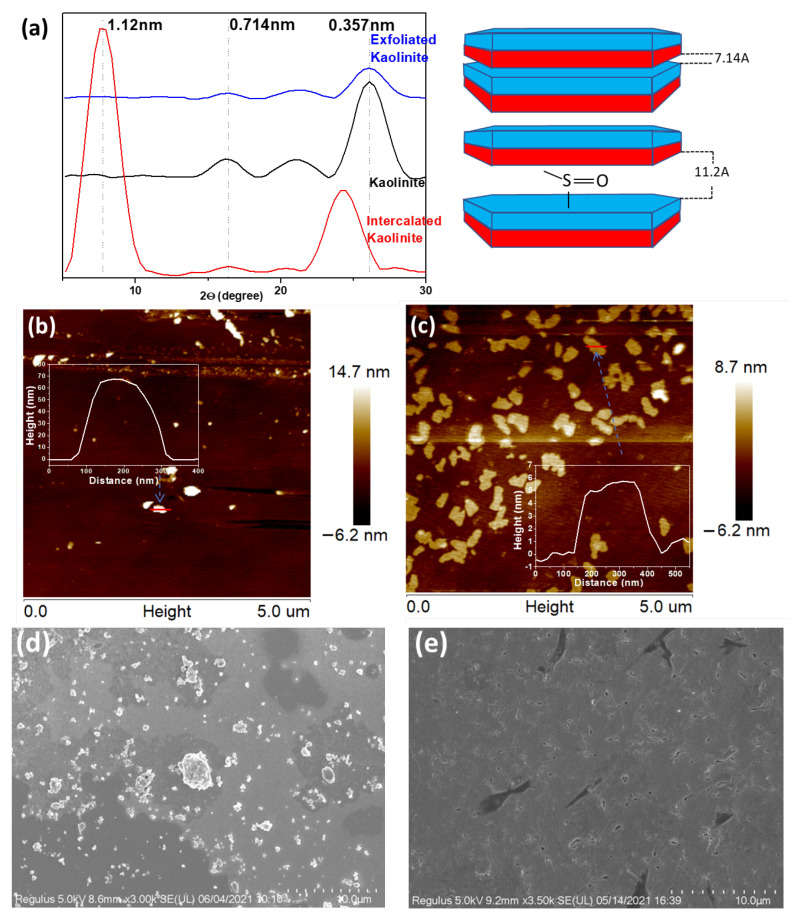
(**a**) XRD patterns of pristine, DMSO intercalated, and exfoliated kaolinite; (**b**,**c**) AFM images and height profiles of pristine kaolinite (**b**) and exfoliated kaolinite nanosheets (**c**) deposited on fresh silicon wafer; (**d**,**e**) SEM images of pristine kaolinite (**d**) and exfoliated kaolinite nanosheets (**e**).

Figure 1b schemes the process of the amphiphilic Janus nanosheets preparation from pristine kaolinite via dimethyl sulfoxide (DMSO) intercalation and subsequent exfoliation facilitated by sonication before being grafted with the silane coupling agent KH570 on the AOS side. Note that the further modification with PNIPAM was not schemed in the figure, as that is not the main sample of focus but an additional one with its amphiphilicity modulable to support the demonstration of the role of moderate amphiphilicity on stabilizing emulsions for enhanced EOR. The successful intercalation of DMSO into pristine kaolinite (Kaol) can be verified by the increased interlayer spacing, as identified from XRD measurements (Figure 2a). The shift of the d001 crystal plane from 16.22° (corresponding spacing of 0.741 nm) of Kaol to 7.95° (spacing of 1.12 nm) of intercalated Kaol, with the peak at 16.22° greatly weakened, confirming the successful intercalation of DMSO into the interlayer of Kaol (Figure 2a) [28]. Aside from the appearance of the 1.12 nm reflection, the d002 crystal plane (0.357 nm reflection) also shifts to a lower peak (i.e., a wider reflection) after the intercalation of DMSO. However, after exfoliation, the 0.357 nm reflection shifted to the original place with a reduced intensity, indicating degradation, disruption, and/or the loss of the crystal structure of Kaol [29,30] during the exfoliation process. The 1.12 nm peak, which was originally seen from the intercalated Kaol, disappeared due to the successful separation of the stacked layers.

The successful exfoliation with production of nanosheets, which were visualized as flakes, was verified through AFM imagining (Figure 2b,c) and SEM imaging (Figure 2d,e). In the samples of exfoliated Kaol (KaolNS), a large amount of nanosheets dimensioned hundreds nanometer in width with a thickness of ~5 nm was observed in AFM measurements (Figure 2c), in a great contrast to the high thickness (~70 nm) of the Kaol particles (Figure 2b). Note that the exfoliation of the Kaol that roughly consisted of ~10 layers in an individual particle resulted in a larger number of particles in the KaolNS (Figure 2c), given that their concentration for AFM was the same (~10 ppm). A difference in the morphologies and sizes between the Kaol and KaolNS was also observed in SEM imaging (Figure 2d,e), with the Kaol existing in terms of particles and/or aggregates, whereas the KaolNS formed a smooth film-like coating on the substrate.

In an individual kaolinite nanosheet, the Alumina Octahedral Sheet (AOS) possesses a hydrophilic µ-Al2-OH group, whereas the Silica Tetrahedral Sheet (STS) has exposed oxygen atoms. Hence, the AOS exhibits a much higher reactivity for further chemical modification [23]. That being said, the hydroxyl groups (-OH) present on the AOS play a crucial role in linking the silane coupling agent, the KH570 molecules, to the clay surface by forming an Al-O-Si bond (Figure 1). Note that aside from forming Al-O-Si bonds with clay surface, the excess silanol groups on the hydrolyzed KH-570 silanes can self-condense into -Si-O-Si- networks, as schemed in Figure 1b.

**Figure 3:**
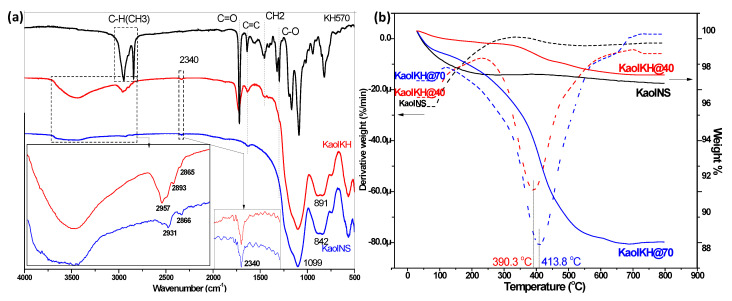
(**a**) FTIR spectra of the KaolKH (red) with KH-570 (black) and KaolNS (blue) presented as reference; (**b**) TGA data of KaolKH@40 (red) and KaolKH@70 (blue) with KaolNS (black) shown as reference.

Figure 3a shows the FTIR spectra of the KH570 modified Kaol (KaolKH) in comparison with KaolNS and KH-570. The 1099 and 842 cm^−1^ bands were assigned to the Si-O and Al-OH of kaolinite, respectively. The inner hydroxyl, which is responsible for the hydrogen bonding of the AOS and STS located in-between the layers, is located at the 2340 cm^−1^ band, whereas the bands of the outer hydroxyls were located from the region 2821 to 3715 cm^−1^ [31]. After the grafting of the KH-570 to KaolNS, the CH_3_ band of the KH-570 located at 2945 and 2841 cm^−1^ was observed as a band in the KaolKH around 2957 cm^−1^, confirming the successful grafting of KH-570 to the nanoparticle. Moreover, a change in band area was observed for the outer hydroxyl band around 3440 cm^−1^ of the KaolNS after the grafting with KH-570, whereas the inner hydroxyl remained unaffected after the silanization process, showing that no hydrogen bonding occurred with the said hydroxyl group, as it was recessed in-between the AOS and STS; thus, they were not influenced by any modifications due to their orientations towards the vacant sites [32]. Furthermore, the bands of the nonhydrolyzable alkyl groups of the KH-570, otherwise labeled as group R, were seen as grafted to the nanoparticles as shown in the 1720, 1638, 1454, and 1296 cm^−1^ bands of C=O, C=C, CH_2_, and C-O, respectively, of the KH-570, which were then visible on the KaolKH’s FTIR spectrum at 1724, 1637, 1455, 1321, and 1295 cm^−1^. As modifications took place on the AOS side, a slight band shift of Al-OH from 842 to 891 cm^−1^ was also observed.

TGA measurement was performed to estimate the amount of KH-570 grafted to the KaolNS, with the data of weight loss and derivative weight loss of KaolNS and the two KaolKHs (i.e., KaolKH@40 and KaolKH@70) shown in Figure 3b. The initial mass loss observed from 30 °C to 150 °C in the three samples was a result of dehydration [33], and, as no oven heating was performed with the samples prior to the TGA analysis, the mass loss for dehydration varied. The weight loss observed from 300 °C to 800 °C, as referenced with the DTG curves, was attributed to the degradation process of KH-570. The peaks maximized at 390.3 °C and 413.8 °C for KaolKH@40 and KaolKH@70, respectively, whereas no evident degradation was observed in KaolNS. Hence, the comparison between modified kaolinites and KaolNS in terms of mass loss from 300 °C to 700 °C would indicate the amount of KH-570 grafted to the KaolNS, and it was calculated that there was a 1.22% difference for KaolKH@40 and a 7.81% difference for KaolKH@70 in comparison to KaolNS, showing that a higher grafting temperature resulted in a higher graft yield. Another study also observed that KH-570 degraded at the same range [34]. Note that though KaolNS appeared to have a lower residual than KaolKH@40 (Figure 3b), the weight loss of KaolNS mostly came from dehydration, while the latter resulted from the degradation of KH570.

**Figure 4:**
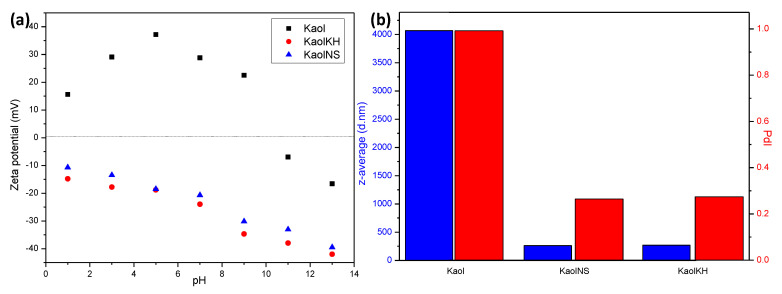
(**a**) Zeta potential and (**b**) particle size and polydispersity index (PdI) of Kaol, KaolNS, and KaolKH particles. KaolKH@70 was used as an example of KaolKH.

As shown in Figure 4a, Kaol owns a positive zeta potential, peaking at 37.2 mV around pH 5 and showing an isoelectric point (iep) around pH 10 before becoming negatively charged. Such a trend has also been reported in the literature [35] and was considered to be general for minerals, especially when hydrolysable metal ions are present, which is possible, in our case, that Kaol contains counter ions within the interlayer. It was argued that the zeta potential becomes more positive as the concentration of hydrolysable ions increases and can become largest at intermediate pH wherein precipitation of hydrolysable metals occurs as metal oxides [36]. Under alkaline pH conditions, the measured zeta potential belongs to the metal-hydroxide-covered solid, being negative. After exfoliation, no matter whether grafted or not with KH-570 (KaolNS and KaolKH), the particles were negatively charged, with an increase in the absolute value of zeta potential with increased pH when pH increased from pH1 to pH13. This may have been due to the protonation and de-protonation effect of the particle surfaces.

Aside from the AFM measurements, the light scattering verified the extremely broad size distribution of particles of Kaol by attaining a PdI value of 1.00, whereas the PdI values attained for both KaolNS and KaolKH were below 0.30, indicating a relatively narrow-sized distribution of particles (Figure 4b). The slight increase in the z-average (in d.nm) and PdI of the KaolKH from the KaolNS was correlated to the presence of the hydrophobic tails. Additionally, a 93.36% decrease was seen in the z-average after the exfoliation of Kaol, indicating the successful exfoliation of the particles into a smaller particle size.

**Figure 5:**
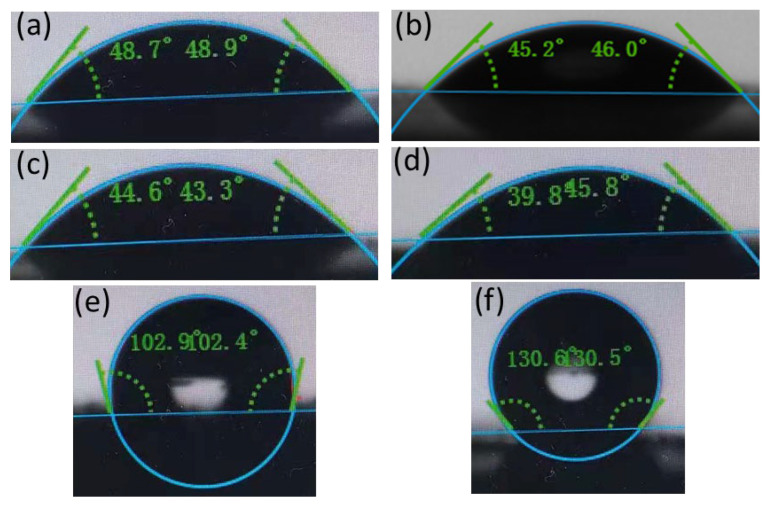
Water contact angle of Kaol (**a**), KaolNS (**b**), and the hydrophilic face (STS face) (**c**,**d**) and hydrophobic face (AOS face) (**e**,**f**) of KaolKH@40 (**c**,**e**) and KaolKH@70 (**d**,**f**).

To verify the amphiphilic and Janus feature of KaolKH, wettability on both sides of the nanosheet was measured in terms of water contact angle, with data shown in Figure 5. When 1 µL of water droplet was deposited on the surface of a clean glass slide, a contact angle of ~60.2° was observed indicating a hydrophilic surface (Figure S1). For Kaol and KaolNS, the contact angle was found to be just at the range of 45.2°–48.7°, indicating that there were roughly no changes on the hydrophilicity made with the side being measured. The contact angles measured based on the interfacial films of the KaolKH@40 and KaolKH@70 showed a distinct difference in wettability between the two faces of the KaolKH nanosheets, with the hydrophilic side (STS face) having a contact angle ~44.6° for KaolKH@40 and ~39.8° for KaolKH@70, whereas the hydrophobic side (AOS face) had a contact angle ~102.9° for KaolKH@40 and ~130.6° for KaolKH@70. This result verified the Janus and amphiphilic nature of the KaolKH nanosheets. Additionally, the AOS face showed a greater hydrophobicity in KaolKH@70 than KaolKH@40, indicating that a higher graft yield of KH570 resulted in a higher hydrophobicity (Figure 3c). A similar increment in hydrophobicity with increased grafted KH-570 on Nano-SiO_2_ was reported in the literature [37].

### 3.2. Interfacial Behavior of Nanosheets in Oil–Water System

#### 3.2.1. Interfacial Behavior Observations

To imitate the hydrophilic rock surface environment in an oil reservoir, a glass tube (hydrophilic inner surface) was used to investigate the interfacial behavior of kaolinite nanosheets in toluene–brine system. In a pure toluene and brine system, a clear concave meniscus was observed at the interface, confirming that the tube surface was preferentially wetted by the aqueous (water-wet) phase (Figure S2a). For pristine Kaol dispersed at 0.5 wt% in the brine phase, even after tip sonication for 5 min, no evident signs of emulsion were observed, with most of the particles aggregated and settling down with time, having only a few of patches lying at the interface towards the side of the lightly cloudy oil phase (Figure S2b,c). The patches were identified under a microscope to be mixtures of particle films and Pickering emulsion droplets (Figure S2d), which, however, quickly demulsified in seconds. The low emulsification ability and instability of the emulsion must have been associated with the large particle size and low surface activity of the pristine Kaol, though there were studies reporting some smaller Kaol particles capable of performing emulsification after certain modifications [38,39].

**Figure 6:**
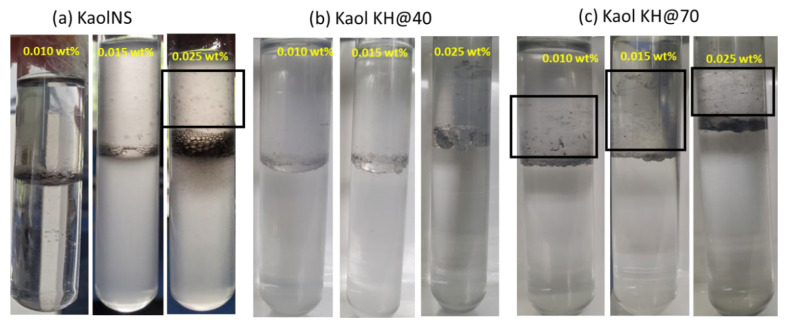
Observation of behavior of nanosheets ((**a**). KaolNS; (**b**). KaolKH@40; (**c**). KaolKH@70) at toluene/brine interface with varied particle concentrations (from left to right, 0.010, 0.015, 0.025 wt%) as prepared in glass tube. The observed climbing films are boxed.

The exfoliated kaolinite (KaolNS), a Janus nanosheet, thanks to the larger surface area and lower effect of gravity that was in favor for surface activity, was able to form emulsions (Figure 6a) when subjected to a 30-s tip sonication, showing a typical Pickering effect. The emulsion was confirmed to be an oil in water (O/W) type, as the conductivity was measured to be above 80 μS/cm (Figure S3A,B). The O/W emulsion height was increased with increased KaolNS concentration. This was under expectation for a nanoparticle with hydrophilic features in forming Pickering emulsion. In addition to the produced emulsions, one interesting phenomenon observed for the KaolNS was the appearance of films climbing upwards the tube surface on the oil side when the particle concentration used was above a critical value (c.a. 0.015 wt%).

For the amphiphilic Janus nanosheets KaolKH@40 and KaolKH@70, having the AOS side of the nanosheet modified with KH-570 to different extents, some differences have been observed regarding the interfacial phenomena at the oil–water interface (Figure 6b,c). For KaolKH@40 that had an intermediate amphiphilicity with a contact angle difference between the AOS and STS side of the nanosheet (∆Θ) being ~58° (Figure 5), O/W Pickering emulsions were produced. The emulsion type was confirmed by dyeing the aqueous phases before observation under microscope. For the KaolKH@70 that had a strong amphiphilicity, with a ∆Θ being ~90° (Figure 5), macroscopically observable interfacial films were formed at the oil–water interface, and the climbing films were also formed upwards on the oil side (Figure 6c), even at a particle concentration as low as 0.01 wt%. After the interfacial film formation, increasing the nanosheet concentration can result in the formation of few O/W Pickering emulsions on the top of the interfacial film. For clarity purpose, the interfacial film was vertically viewed from the top of the tube and under a microscope (Figure S4). As shown, the interfacial film had a wrinkled and bumpy morphology, and the excess nanosheet could be seen with a darker color than the rest of the film.

**Figure 7:**
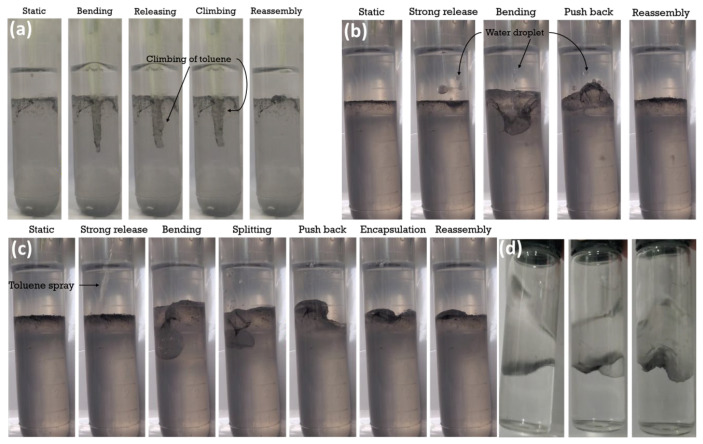
Apparent testing of the elasticity of the interfacial film formed by KaolKH@70 nanosheets at oil–water interface: (**a**) poking with a plastic pipette; aggressively releasing water droplet (**b**) and toluene droplet (**c**) towards the interfacial film; (**d**) gentle shaking with a wave-like motion observed.

Moreover, the interfacial film was demonstrated to be strongly elastic. To intuitively test the elasticity of the interfacial film, a plastic pipette was used to press the interfacial film to almost half the height of the aqueous phase. The interfacial film was still intact, encapsulating the pipette (Figure 7a). After the pipette was retracted, there were no signs of rupturing, as the interfacial film quickly reassembled. Moreover, when one droplet of water was aggressively released towards the interfacial film (Figure 7b), the water droplet was observed to bounce back from the interfacial film before penetrating the film into the aqueous phase after its second contact with the film. When one droplet of toluene was aggressively released towards the interfacial film (Figure 7c), in light of the hydrophobicity on the top surface of the interfacial film, the droplet bended and split the interfacial film, but with the droplet always stuck to the film until fully encapsulated. At the same time, the split films dragged some water to the encapsulation when the films recovered to the planar interface. This indicated that when there were excess nanosheets at the interface or when the interfacial film was oversaturated. Pickering emulsions (most likely multiple emulsions) were possible to be formed on top of or beneath the interfacial films (Figure 6). In addition, as shown in Figure 7d, when shaken side to side, the interfacial film produced a wave-like motion to maintain equilibrium. The wave-motion was inhibited by the system due to the inclusion of the interfacial film acting as the barrier between the two fluids, balancing the interfacial tension from forming droplets or maintaining a concave interface between liquids. The motion was due to the steady redistribution of the nanosheets in the interface because of the acting Marangoni stresses. This characteristic showcased the strong elasticity of the interfacial film by maintaining as anchored at the interface with irregular shapes, different from the concave interface observed in a pure oil–water system that was dominated by the interfacial tension.

#### 3.2.2. Rheology of the Interfacial Film

**Figure 8:**
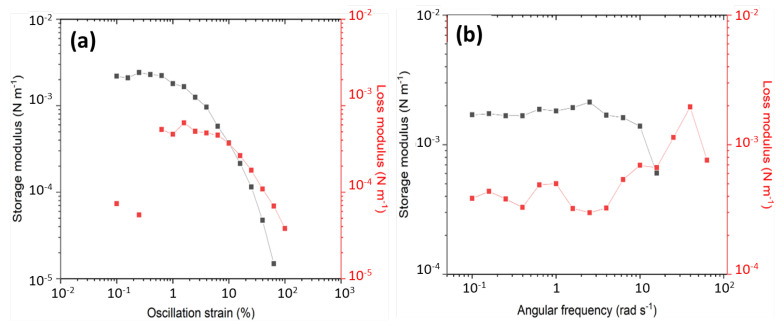
(**a**) Strain amplitude sweep and (**b**) dynamic frequency sweep of the interfacial film formed by KaolKH@70 nanosheets at oil–water interface.

The interfacial film was further characterized using an interfacial rheology measurement. Strain amplitude sweep (Figure 8a) was performed first to determine the linear viscoelastic (LVE) regime. As shown, the interfacial film appeared to have a solid-like structure with a viscoelastic solid behavior dominated by elasticity at low strains, having a greater interfacial storage modulus (G′) than loss modulus (G″), before deviating from the LVE region and transitioning into liquid-like behavior when the strain increased across a value roughly around 10%. The dynamic frequency sweep (Figure 8b) also confirmed that the interfacial film/particles were strongly associated or had a well-structured system, with a G′ value laying above 0.001 N m^−1^, and both had the moduli (G′, G″), being almost independent of frequency, implying a soft-glassy characteristic of the interfacial film [40]. The measurement thus confirmed the good elasticity and stability of the interfacial film, being able to be maintained for a long period during storage.

#### 3.2.3. Possible Mechanism for the Formation of Interfacial Film and Climbing Film on Tube Surface

Evidently, for a given container’s wall–surface wettability, the interfacial phenomenon of the Janus nanosheets at the oil–water interface, whether to produce pure Pickering emulsions or to form observable interfacial films, was dependent on surface chemistry of the nanosheets. A more amphiphilic feature with more KH-570 grafted to the AOS face of the Janus nanosheet favored the formation of a strongly elastic interfacial film.

In fact, formation of the interfacial film of particles at the interface between oil and water phase was a result of failure of the circular droplets’ stabilization by particles during the homogenization process. To illustrate this, the rupturing of an emulsion produced by KaolKH@40 was performed. A toluene–nanosheet–brine system containing 0.020 wt% of KaolKH@40 was repeatedly shaken until it produced an emulsion instead of an interfacial film. The emulsion was then poked with a plastic pipette. As the emulsion that was poked ruptured, the other emulsions also ruptured. These events, which resulted in the collapse of the emulsions, were responsible for the formation of an interfacial film (see Figure S5).

Having an amphiphilicity feature, the Janus nanosheet exhibited a very similar behavior with the surfactants, i.e., tending to reside at the oil (air)/water interface to reduce the interfacial tension between the two phases at low concentrations before being able to form emulsions when the concentration was in excess, above its critical micelles concentration (CMC), more exactly. The only difference was that the interfacial films formed by the Janus nanosheets were eye-observable and strongly elastic but not for the surfactant-based interfacial films. The critical particle concentration to form the interfacial films could be associated with the tube’s size. It was shown that the smaller the tube size the smaller number of particles needed to form interfacial films (see Figure S6), indicating the importance of the meniscus force (i.e., Laplace pressure) in dictating film formation.

**Figure 9:**
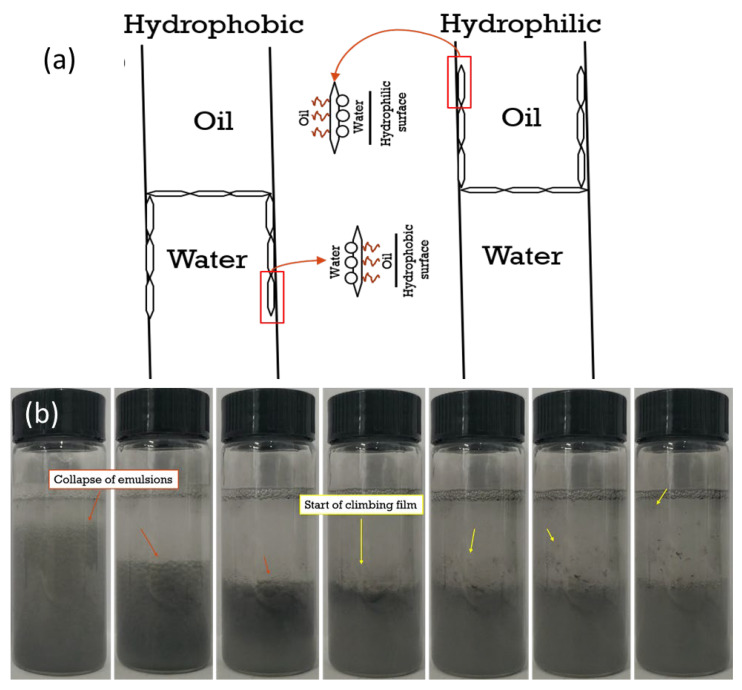
(**a**) The proposed schematic, illustrating the climbing films observed on hydrophobic and hydrophilic container surfaces; (**b**) the formation of climbing film as a result of the collapse of emulsions after shaking a toluene-brine (17 mM) system containing a 0.035 wt% KaolKH@70.

Moreover, the stabilization of droplets by Janus nanosheets during the emulsion generation was greatly affected by the particle–container surface interactions. Upon droplet generation, there was a competition between the tendency of the nanosheets to stabilize the droplet and the tendency of the nanosheets to adhere to the tube’s surface, which could be somewhat analogous to the superspreading of the surfactant-coated drop on the substrate. According to Hsuen-Hung Wei [41], some surface-active agents can undergo a phenomenon called the “local surfactant leak”, wherein the surfactants’ hydrophilic heads are attached to hydrophilic solid surface instead of the aqueous phase or that the surfactants’ hydrophobic tails are attached to hydrophobic surface instead of the oil phase. The adherence of the nanosheets to the container wall’s surface resulted in the clearly observed climbing films, upwards on the oil side in a hydrophilic container (Figure 6) and downwards on the water side in a hydrophobic container (Figure S7), respectively. This phenomenon was termed “Marangoni-enhanced capillary wetting”. It was also reported that the climbing film had a sandwich structure, wherein the particles were present between the oil and water phases (Figure 9a) [42].

Moreover, the formation of climbing films could have been resulted from the emulsion destabilization if the tendency of the particle–surface interaction was dominant over its ability of stabilizing the droplets. Figure 9b shows the formation process of the climbing films as a result of emulsion destabilization when subjected to rigorous shaking. This was consistent with an early study of Binks et al. [43], who mentioned the formation of climbing films as a consequence of the collapse of emulsions.

#### 3.2.4. Reversibility between Stable Emulsion and Interfacial Film

Furthermore, to evaluate the possibility of transition between the observable interfacial films and the stable emulsions, the Janus kaolinite nanosheets were made to be temperature responsive by grafting with a PNIPAAm that had a lower critical solution temperature (LCST) of ~32 °C, a method typically used in the literature [44]. In particular, the KaolKH@70 that had KH570 grafted on the AOS side of the nanosheet was further polymerized with N-Isopropylacrylamide, to achieve the “smart” Janus nanosheet (i.e., KaolNP@70). Figure S8 shows the TGA data of KaolNP (i.e., KaolNP@70) using pure PNIPAAm as a reference. As shown, there was a significant mass loss in the range between 300 °C and 500 °C, which was attributed to the degradation of the organic components, including PNIPAAm and KH570. The PNIPAM started degradation above 300 °C, as reported in literature [45,46], in a similar range with the KH570. By subtracting the KH570 amount determined in the KaolKH@70 (Figure 3b), the amount of PNIPAAm being grafted in the KaolNP was determined to be ~17.82%. Through having both sides of the nanosheets be hydrophilic at temperatures below the LCST of the PNIPAAm, while one side was hydrophilic and the other was hydrophobic above the LCST, the KaolNP was expected to form thermo-responsive emulsions that could be stabilized and destroyed at will.

**Figure 10:**
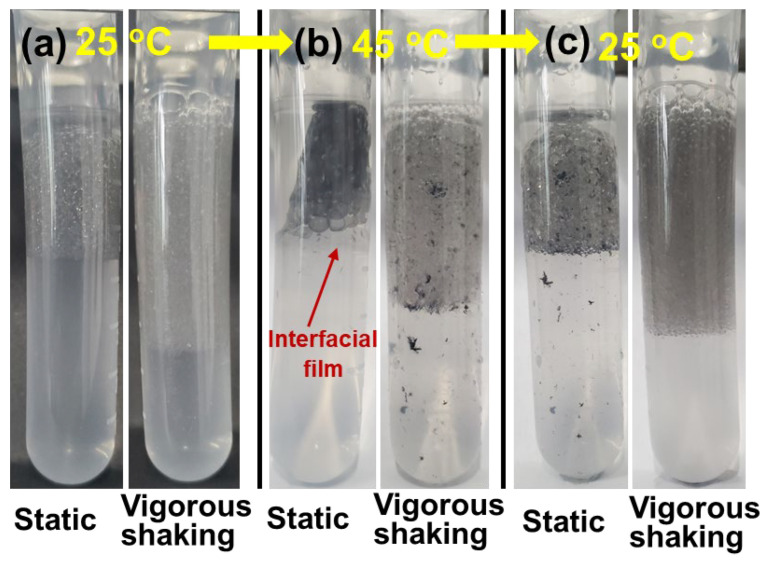
The observations of the oil–nanosheet–water system containing 0.06 wt% KaolNP@70 in static and vigorous shaking states: (**a**) after sonication at room temperature (25 °C), (**b**) subjected to heating at 45 °C in water bath for 5 min, then (**c**) being cooled back to room temperature.

Upon subjecting to tip sonication at room temperature (i.e., 25 °C), oil-in-water (O/W) Pickering emulsions were produced at the toluene/brine interface when the KaolNP (0.06 wt%) was present (Figure 10a), different from the case of KaolKH@70 that formed eye-observable interfacial films in both hydrophobic plastic (Figure S7) and hydrophilic glass (Figure 6) tubes. However, when the temperature was increased to 45 °C, the O/W Pickering emulsions gradually shifted to the formation of eye-observable interfacial films with some mixed multiple Pickering emulsions (Figure 10b). This was in agreement with the previous results observed with KaolKH@70, where the AOS side was hydrophobic, and solid interfacial films were formed (Figure 6). After cooling down to room temperature and then shaking, the formation of stable Pickering emulsions was again observed, and no interfacial film was present (Figure 10c). This indicated that the hydrophilic KaolNP nanosheets were surface active and able to form emulsions similar to the KaolNS and the KaolKH@40 (Figure 6) below the LCST of the PNIPAAm, whereas, above LCST, the PNIPAAm chains on the AOS side of the KaolNP became hydrophobic, and the amphiphilic nanosheets showed a similar behavior to KaolKH@70, being able to form eye-observable interfacial films. Moreover, the transition between the Pickering emulsions at 25 °C and the formation of interfacial films at 45 °C was reversible.

**Figure 11:**
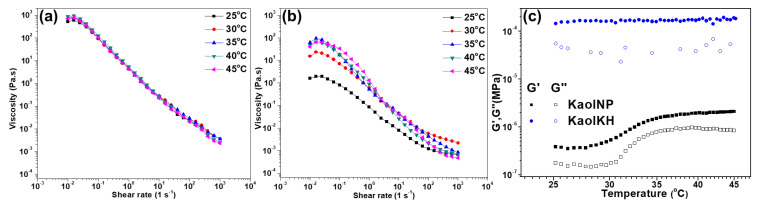
The steady shear flow curve at different temperatures of the emulsions stabilized by (**a**) KaolKH and (**b**) KaolNP and (**c**) their dynamic shear moduli obtained via temperature ramping from 25 °C to 45 °C.

The emulsions stabilized by KaolNP were subjected to rheological measurements in terms of steady shear and dynamic shear to evaluate the temperature effects, with those of KaolKH used as reference. As shown in Figure 11a,b, both emulsions of KaolNP and KaolKH showed shear-thinning behaviors but showed differences in temperature dependence within the range of 25 °C to 45 °C. Different from KaolKH, which showed no temperature dependence in the emulsion viscosity. The KaolNP showed clear temperature dependence, with its viscosity increased as the temperature was increased. Additionally, the temperature ramp test showed that the emulsions of the KaolNP experienced an abrupt increase in both moduli (storage modulus G′ and loss modulus G″) within the temperature range around the LCST (~32 °C), in great contrast to the steady state of the emulsions of the KaolKH (Figure 11c). This further confirmed the temperature responsiveness of the emulsions stabilized by KaolNP, being stable at low temperatures while collapsed into solid interfacial films at high temperatures above the LCST of PNIPAAm, in the range of 30 °C to 35 °C.

### 3.3. Enhanced Oil Recovery Efficiency of KaolKH Nanofluids

#### 3.3.1. Core Flooding Results

To determine the potential of using the nanofluids of kaolinite-based Janus nanosheets for enhanced oil recovery, oil displacement experiments were conducted based on a hydrophilic core of sandstone. Nanofluids of KaolKH@40 and KaolKH@70, with those of KaolNS and Kaol used as references, were evaluated at a concentration of 0.01 wt% using four individual cores. The rock porosity of the core was measured to be about 40%, and the permeability data of the cores are listed in Table 1. The viscosity of the used crude oil was measured as a function of temperature (data shown in Figure S9), being 95 mP.s at the temperature (25 °C) used for the core flooding test.

**Figure 12:**
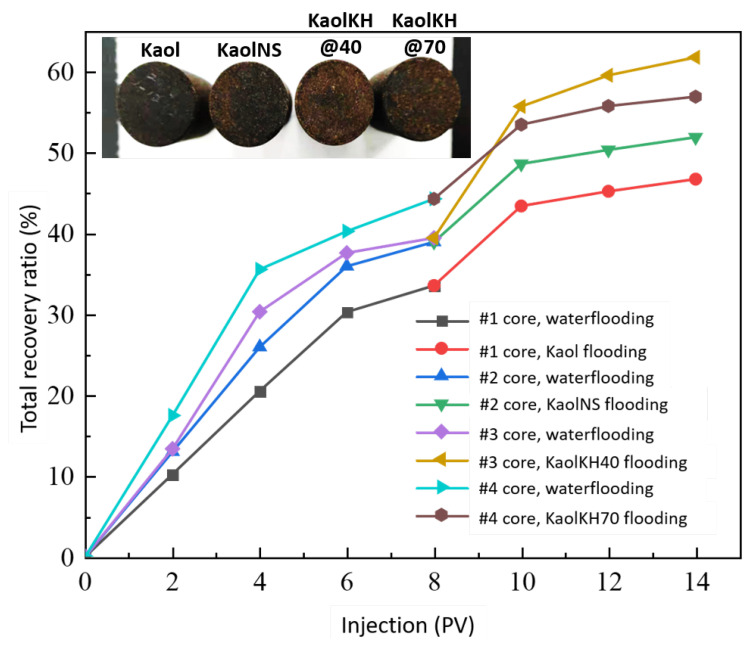
The results of the core flooding test by Kaol, KaolsNS, KaolKH@40, and KaolKH@70. All tests were performed at 25 °C.

Figure 12 shows the oil recovery versus pore volume (PV) of the fluid injected for conventional (brine flooding) and EOR (nanofluid flooding) processes. All the effluents, including the recovered oil and flooding fluids, were collected using a volumetric cylinder (see Figure S10). After injecting 8.0 PV of brine solution for each experiment, around 33–44% of the oil was recovered relative to the original oil in place (OOIP), meaning that around 56–67% of the OOIP remained unrecovered by the means of conventional water flooding. Subsequently, after 3.0 PV of nanofluid flooding, the enhanced oil recovery was around 12.6–13.2% for Kaol, KaolNS, and KaolKH@70, whereas it was 22.37% for KaolKH@40, indicating an enhancement on the EOR rate via the KH570’s functionalization on the Kaol nanosheets at 40 °C. The insets of Figure 12 are the cores after brine and nanofluid flooding. As the recovery rates from waterflooding are included, the Kaol’s core had the most residual oil saturation with KaolKH@40 having the least. As compared to the literature, the EOR efficiency of the KaolKH@40 (i.e., 22.37% at 0.01 wt% nanosheets) obtained here was strongly superior to the performance of the homogeneous nanoparticles (e.g., an average EOR factor ~4% of 0.01 wt% SiO_2_ nanofluids and 5% of 0.3 wt% Al_2_O_3_ nanofluids) [4]. The efficiency was also greater than nanofluids of other types of Janus nanoparticles, for example, an EOR factor ~15.2% of 0.01 wt% graphene-based amphiphilic Janus nanofluids [12], 15.74% of 0.01 wt% SiO_2_-C1_2_ Janus nanofluid [47], ~18.31% of 0.005 wt% carboxyl/alkyl composite silica-based amphiphilic Janus nanosheets fluids [14], and ~21% of 0.25 wt% natural halloysites-based Janus platelet nanofluid [48].

#### 3.3.2. Potential EOR Mechanisms

**Figure 13:**
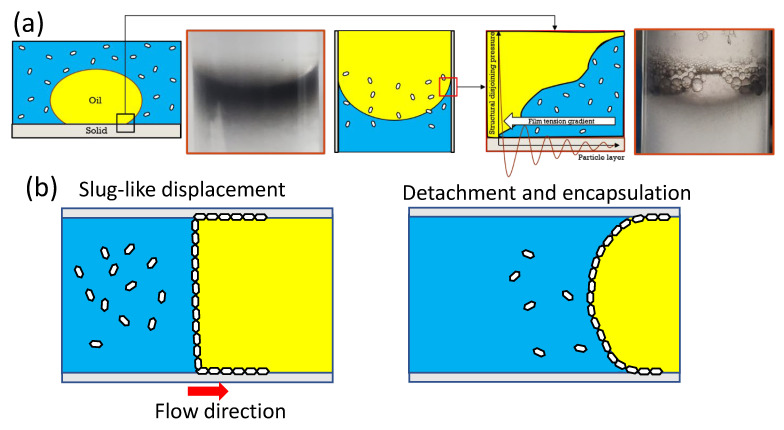
(**a**) The similarity of an oil droplet’s three-phase contact with a concave meniscus; (**b**) reported mechanism of interfacial films and/or climbing films.

Both the formation of Pickering emulsions and the observable interfacial films together with climbing films produced from nanofluids have been reported to be favorable for enhanced oil recovery (EOR). According to the proposed oil displacement presented by Wasan and Nikolov [49], a pressure known as structural disjoining pressure occurred when a nanoparticle was used to displace oil. The nanoparticles formed a layered structure at the three-phase contact region, wherein the structural disjoining pressure occurred. This was performed by the nanoparticles structuring at the wedge of the oil droplet. The disjoining pressure was strongest if the particle layer was smallest [50]. As the nanoparticles approached the wedge, the structural disjoining pressure was at its peak (Figure 13a). As the nanoparticles continued pushing, the oil droplet became less attached to the surface and eventually detached, thus allowing the nanoparticles to form an emulsion. The formation of Pickering emulsions by the nanofluids in glass tubes in the current study demonstrated a similarity to the formation of emulsions accompanying oil displacement from rock surfaces, as driven by the disjoining pressure (Figure 13a). Hence, it was vital to have the formed Pickering emulsions to be as stable as possible.

On the other hand, the planar interfacial films and climbing films have been reported by Luo et al. to favor oil displacement, and the mechanism was argued to be either a slug-like displacement by the interfacial film (Figure 13b-left) or an encapsulation of climbing films over the oil phase, resulting in its displacement from the solid surface (Figure 13b-right). In fact, the later mechanism was much more associated with the structural disjoining pressure, driven by the particle concentration gradient of transferring the particles towards the three-phase region. Hence, its efficiency may have relied on the stability of the following O/W emulsions stabilized by the particles.

In the current study, as demonstrated above, all the nanofluids have shown certain EOR efficiencies, with the KaolKH@40 exhibiting the best EOR efficiency, about 10% higher than the others (i.e., Kaol, KaolNS, KaolKH@70), which have recovery rates of around 13%. The KaolKH@40, Kaol, and KaolNS nanosheets all formed Pickering emulsions in glass tubes, where the solid surface was hydrophilic, similar to the core rock’s surface. Kaol and KaolNS, both surface active, despite forming Pickering emulsions, performed less effectively than KaolKH@40 and roughly gave the same nanofluid flooding recovery rate. This was due to the Kaol not being able to stabilize emulsions for a long time, as shown in Figure S1, wherein emulsions, as observed under a microscope, quickly demulsified. For KaolNS, as shown in Figure 6a, its presence of climbing films was, according to Binks et al. [43], a consequence of the collapse of emulsions. This case was not evidently observed for KaolKH@40, thus showing that the emulsions were stable. Its better oil recovery performance, being 10% higher than those that were unmodified, indicated the importance of the stable emulsions produced by the surface-active and amphiphilic KaolKH@40 Janus nanosheets. The role of emulsion in improving the oil recovery factor was believed to be either through the reduction in IFT, the reduction in the oil’s viscosity, or by improving the mobility ratio [51].

KaolKH@70, which was modified with more KH570 to have a greater amphiphilicity than KaolKH@40, preferred to form interfacial films and climbing films along a hydrophilic surface instead of stable emulsions. This was mainly due to the great difference in the wettability between the two faces of the plate that allowed the plate to adhere more readily to the solid surface. The better emulsion stability of the KaolKH@40 than the KaolKH@70 could also be witnessed from a 3-month aging test (see Figure S11). As shown, the KaolKH@40’s Pickering emulsions were partially collapsed into aggregates, forming some climbing films, but still leaving behind some stable emulsions after 3 months. In the case of the interfacial film formed by the KaolKH@70, after 3 months, no evident changes were observed, showing the great stability of the interfacial films, having no emulsions.

Even if the elastic interfacial films played some roles in the EOR, the deficiencies of the emulsions in the KaolKH@70 resulted in it showing a relatively weak EOR performance, a recovery rate value as low as the Kaol and KaolNS. This, again, demonstrated that the instability of emulsions, despite being unable to be stabilized by particles at the very beginning or the collapse during the shaking to form climbing films/interfacial films, was unfavorable for the EOR. The ability of KaolKH@40 to easily transport oil through emulsification allowed it to perform better than KaolKH@70 in core flooding tests. Not only did this show that Pickering emulsions remained a better choice for performing EOR or increasing the oil recovery factor, but it also showed that an interfacial film could also perform as an EOR method but at a lower efficiency. Emulsions could improve the oil recovery factor, in particular, an oil-in-water emulsion could reduce the mobility ratio [52] whereas water-in-oil emulsions could increase the capillary number, as they exhibited a non-Newtonian behavior [53]. The increase in the capillary number, as well as the reduction in the interfacial tension, resulted in an increase in the so-called microscopic displacement efficiency, whereas the reduction in the mobility ratio resulted in an improvement in the macroscopic oil displacement efficiency [54]. In addition, smaller average droplet sizes of emulsions could be attained by increasing the concentrations of the nanosheets (e.g., the KaolKH@40) (see Figure S12), which would prevent any blockages of pores that may cause problems to the extraction of the crude oil.

## 4. Conclusions

In this study, kaolinite was first intercalated with DMSO and exfoliated via ultrasonication into Janus nanosheets (KaolNS), and the exfoliated nanosheets had been well confirmed by AFM. Having the Alumina Octahedral Sheet (AOS) side be more actively grafted with 3-methacryloxypropyl-triemethoxysilane (KH570) than the Silica Tetrahedral Sheet (STS) side, the KaolNS was readily converted to an amphiphilic Janus nanosheet, and the amphiphilicity was dependent on the grafting temperature, with an intermediate amphiphilic Janus nanosheet, KaolKH@40, obtained at 40 °C and a strong amphiphilic Janus nanosheet, KaolKH@70, obtained at 70 °C, respectively. The amphiphilicity and Janus features had been well demonstrated by contact angle measurements that showed the hydrophobicity on the AOS surface and the hydrophilicity on the STS surface of the nanosheets.

When the mixture of toluene and Janus nanosheets containing aqueous dispersions in glass tubes was subjected to ultrasonication, the KaolKH@40 preferentially stabilized oil in water (O/W) emulsions, while the KaolNS and KaolKH@70 tended to form observable planar interfacial films at their oil–water interfaces, as well as films climbing along their respective tube surfaces. The interfacial film was demonstrated to be highly elastic with a high strength. The formation of the interfacial films and climbing films were associated with the failure of emulsification and/or emulsion collapse as a result of the strong adherence of Janus nanosheets towards tube’s surface.

The core flooding tests in a hydrophilic core were able to differentiate the performance of Pickering emulsions formed by KaolKH@40 and the interfacial film formed by KaolKH@70. It was observed that Pickering emulsions remained a better choice for EOR, as they performed ~10% better, with an EOR rate of 22.37% for nanofluids containing 0.01 wt% KaolKH@40, than an interfacial film, but the interfacial films still showed good oil recovery percentages at such low nanosheet concentrations. Overall, nanofluids of an amphiphilic Janus nanosheets have the potential to be applied to oil recovery enhancement at a low particle concentration, especially when they are able to form stable Pickering emulsions. This strategy for EOR showed advantages over traditional nanofluids, at least including low cost, high environmental friendliness, and high efficiency with a low particle concentration.

## Figures and Tables

**Table 1:** Key parameters of core flooding and the respective oil recovery rates of nanofluid flooding.

Core	Core Permeability (mD)	Water Flooding Recovery Rate (%)	Nanofluid Flooding Recovery Rate (%)	Total Recovery Rate (%)
Kaol (#1)	496	33.57	13.18	46.75
KaolNS (#2)	490	38.97	12.98	51.95
KaolKH@40 (#3)	470	39.47	22.37	61.84
KaolKH@70 (#4)	450	44.3	12.67	56.97

## Data Availability

All the data are contained in the article and the supplementary material.

## Supplementary Materials

The following are available online at https://www.mdpi.com/article/10.3390/polym15112515/s1, Figure S1: Water contact angle of glass substrate; Figure S2: Identification of emulsion types prepared from pristine kaolinite; Figure S3: KaolNS, and KaolKH@40; Figure S4: Interfacial films formed at oil–water interface by KaolKH@70; Figure S5: Rupturing of emulsion prepared by KaolKH@40; Figure S6: Effect of tube radius on the formation of interfacial film; Figure S7: Interfacial behavior of KaolKH@70 in hydrophobic plastic tube; Figure S8: TGA data of KaolNP particles and pure PNIPAAm; Figure S9: Viscosity of oil used in core flooding test; Figure S10: The recovered oil; Figure S11: Age effect on emulsion and interfacial film; Figure S12: the effect of KaolKH@40 concentration on emulsion droplet size [55].
