# Supplementary material for: Nanofluids of Amphiphilic Kaolinite-Based Janus Nanosheets for Enhanced Oil Recovery: The Importance of Stable Emulsion
Source: Polymers (Basel). 2023 May 30;15(11):2515. doi: 10.3390/polym15112515 (PMC10255877; doi:10.3390/polym15112515)
Supplement: Supplementary file 1 [file polymers-15-02515-s001.zip › polymers-2405690-supplementary.pdf]

SUPPLEMENTARY MATERIAL

I. Water contact angle of glass substrate

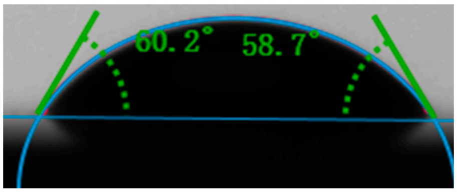

Figure S1. Water contact angle of glass slide

II. Data relevant to the interfacial study

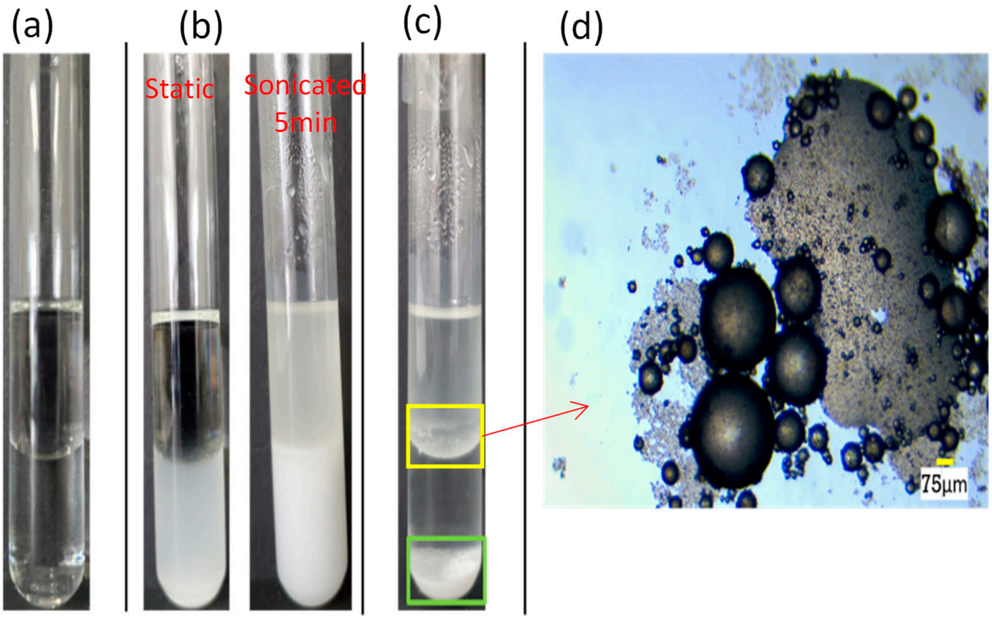

Figure S2. (a) the static toluene/brine (0.1 wt% NaCl solution) interface showing a meniscus curve; (b) observations immediately after 5-minute tip sonication of toluene/brine with pristine kaolinite, showing no obvious sign of IFT reduction ; (c) observations after the system being equilibrated from sonication; and (d) microscopic observation over the patches at the oil/water interface

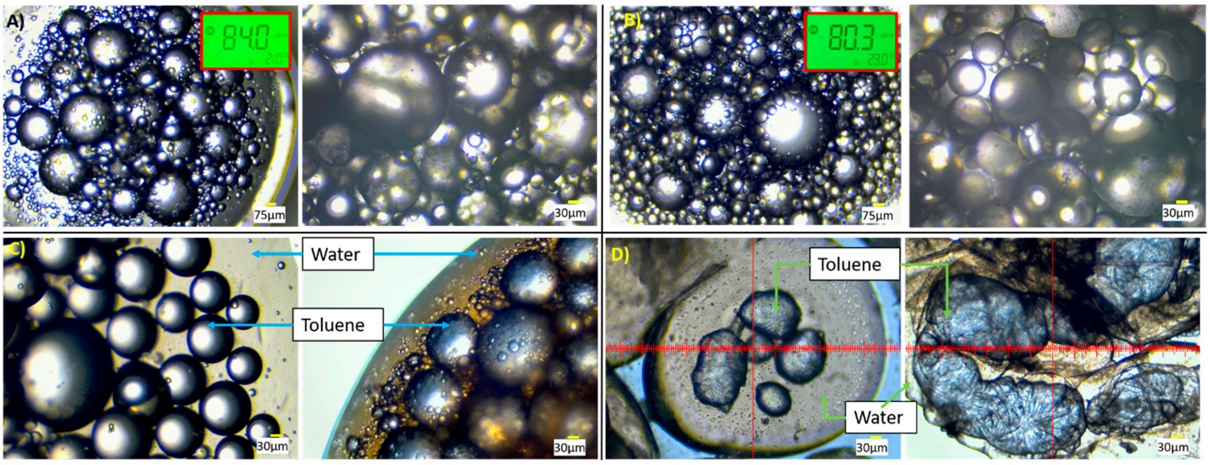

Figure S3. The conductivity of the emulsions formed by KaolNS (A-B), and the identification of the emulsions formed by KaolKH@40 by dyeing the aqueous phase (C-D)

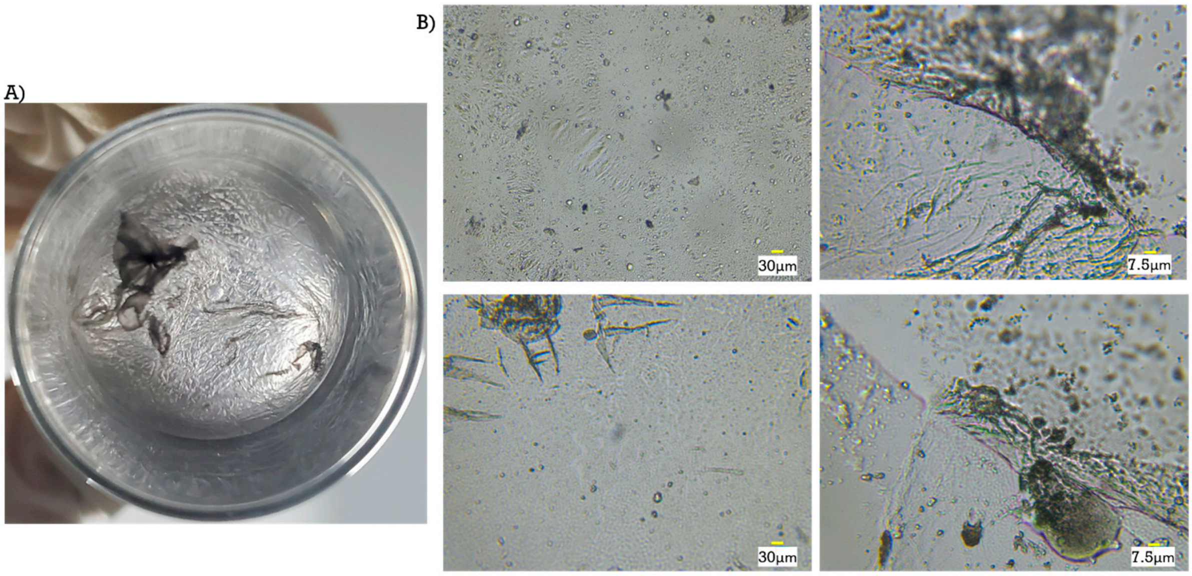

Figure S4. The interfacial film formed by KaolKH@70 (*0.035 wt%*) at the oil/water interface as observed (A) from top of the glass tube having a radius of 27mm and (B) under microscope.

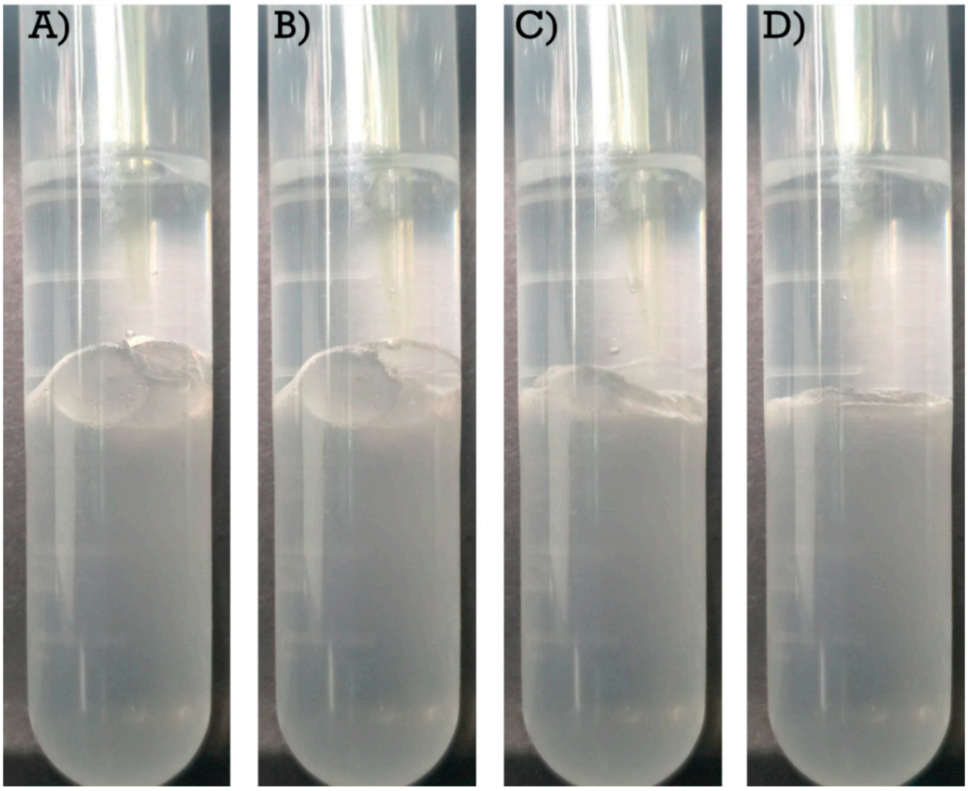

Figure S5. The rupturing of an emulsion to produce an interfacial film. (A) The static emulsions formed by KaolKH@40 (0.020 wt%) was (B) poked with a plastic pipette thus also resulting to the (C) rupturing of the emulsion on the left side of the poked emulsion. The rupturing of the emulsion (D) results to the formation of the interfacial film.

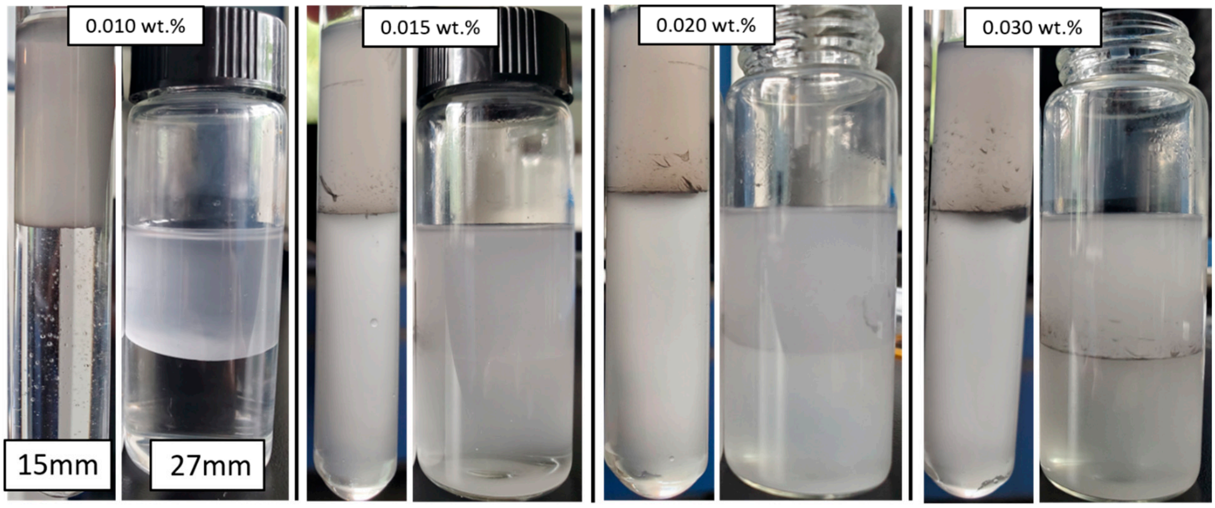

Figure S6. Effect of the radius of the tube on the amount of nanosheets required to form an interfacial film.

According to Y. Hirose et al[1], for an interface, when the curvature increases, for example, realized by reducing tube diameter, it results to an increase of the internal (*Laplace*) pressure which makes it more difficult for the particles to anchor at the interface. Experiments were performed on tubes with different diameters ranging from 15 mm to 27 mm to study the effect of tube radius on the formation of the interfacial film. With a 15mm tube having a tube area of 10,833 mm^2 (*Fig. S6*), the interfacial film was observed in hydrophilic tube with a nanosheet concentration of 0.010 wt% of KaolKH@70 and became more saturated at an increasing concentration. In a 27mm hydrophilic tube having a tube area of 16,871.22 mm^2, the concave meniscus was still present at 0.010 wt% and the concentration of Janus nanosheet where the interfacial film can be observed increased to 0.030 wt.%. An increase of 6 mm on the tube radius, or 6,038.22 mm^2 increase in area, required an increase of nanosheet concentration from 0.010 wt% to 0.030 wt%. This indicates that either the meniscus force (*i.e., Laplace pressure*) or the increase in area that the interfacial film needs to cover plays an important role in the interfacial film formation as an increase of tube radius means a reduction of Laplace pressure and a reduction of curvature of the curved interface.

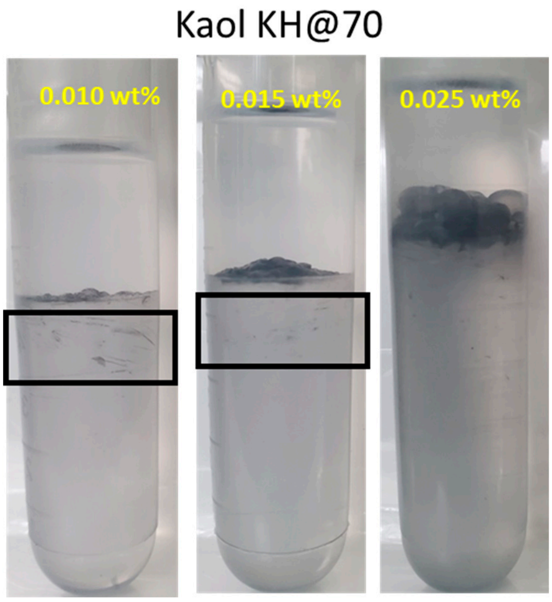

Figure S7. Behavior of KaolKH@70 nanosheets at toluene/brine interface with varied particle concentrations as prepared in plastic tube (hydrophobic wall surface). The observed climbing films are boxed.

III. Data relevant to the thermo-responsive Janus nanosheets

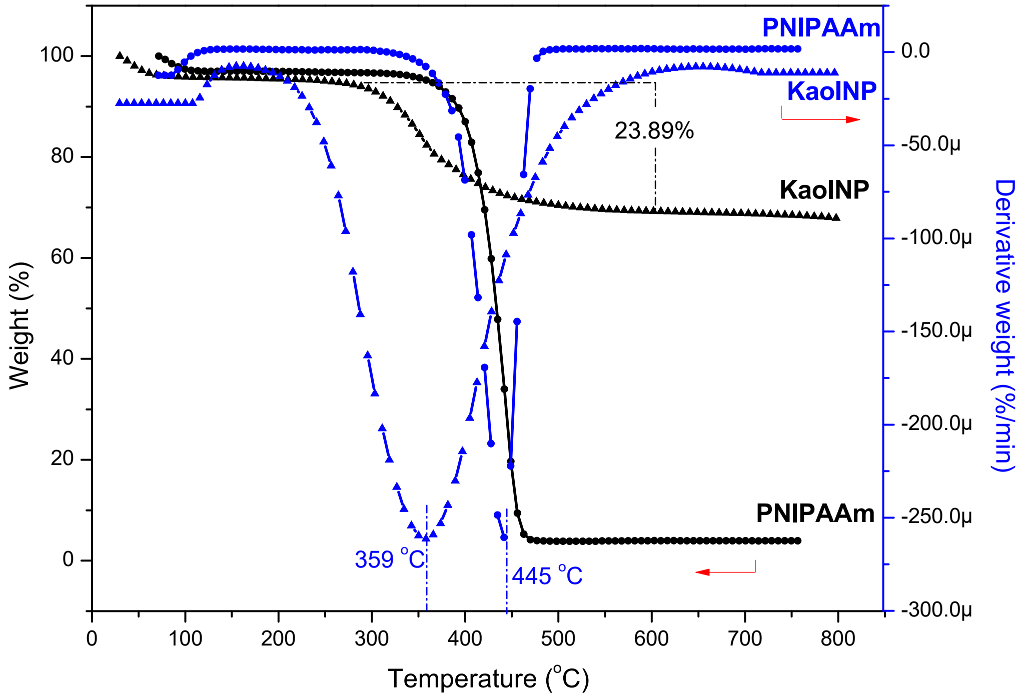

Figure S8. TGA data of KaolNP particles and pure PNIPAAm.

IV. Data relevant to the core flooding

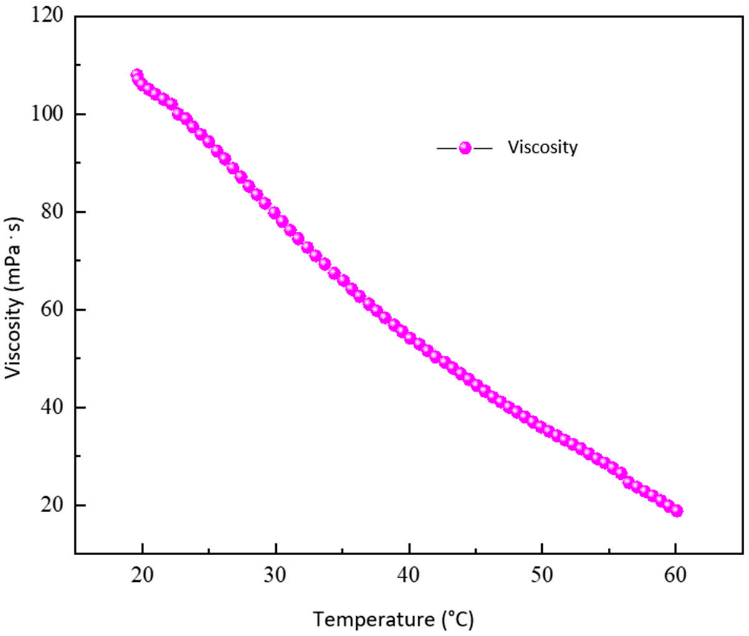

Figure S9. The viscosity of the oil used for core flooding test.

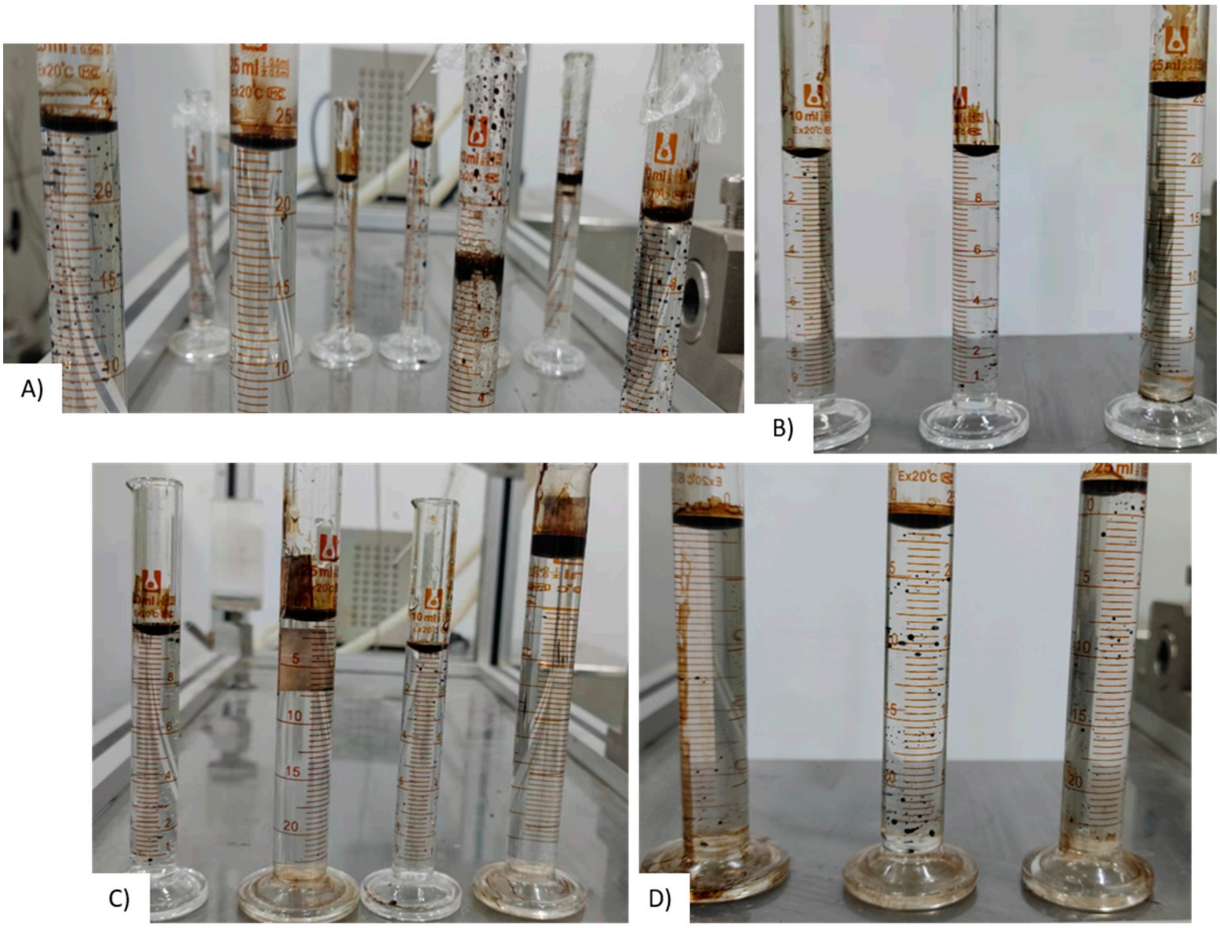

Figure S10. The oil recovered by (A) Kaol, (B) KaolNS, (C) KaolKH@40, and (D) KaolKH@70 after nanofluid flooding.

IV. Other Data for EOR mechanism explanation

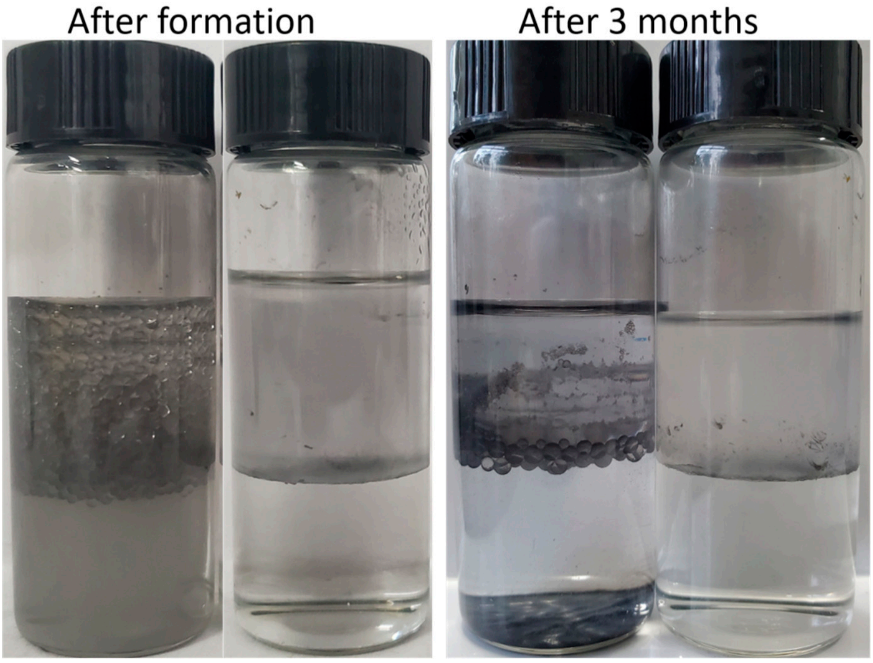

Figure S11. Stability comparison of KaolKH40's Pickering emulsion (left) and KaolKH@70's interfacial film (right), both in 0.30 wt% nanosheet concentration, in a 27mm glass tube.

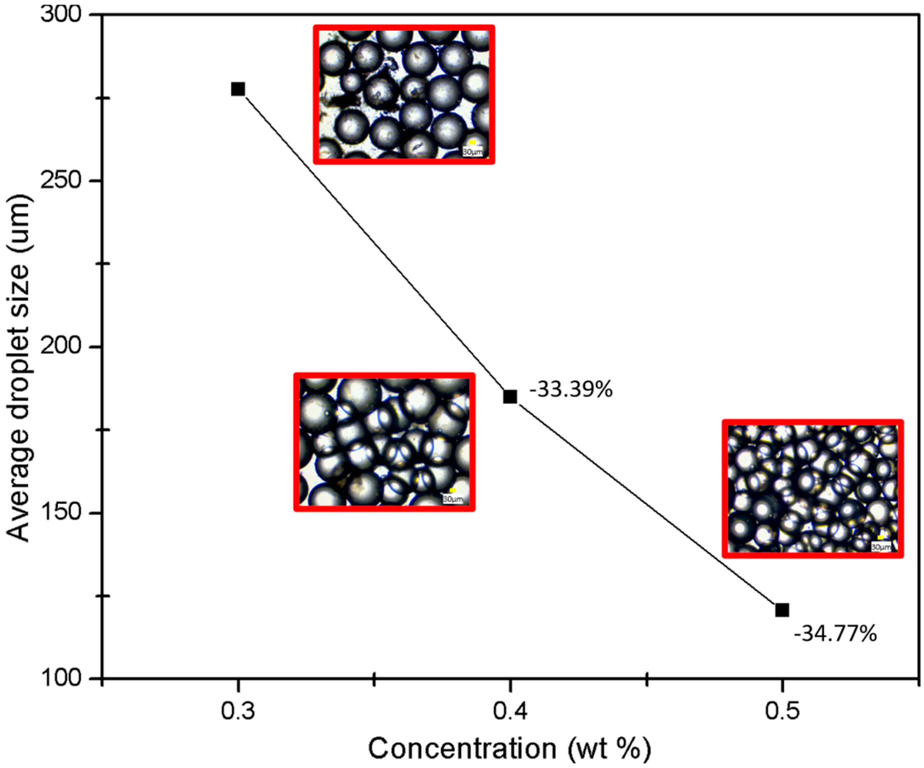

Figure S12. The effect of nanosheet concentration on the average droplet size of emulsions stabilized by KaolKH@40.

References

[1] Hirose, Y., Komura, S., & Nonomura, Y. (2007). "*Adsorption of Janus particles to curved interfaces*". The Journal of Chemical Physics, 127(5), 054707. doi:10.1063/1.2756828
